# GART promotes the proliferation and migration of human non-small cell lung cancer cell lines A549 and H1299 by targeting PAICS-Akt-β-catenin pathway

**DOI:** 10.3389/fonc.2025.1543463

**Published:** 2025-03-25

**Authors:** Zhuo Chen, Yu-Heng Ding, Mei-qi Zhao, Yong-jun Zhang, Meng-Ying Sun, Ai-Qin Zhang, Xiang Qian, Xu-Ming Ji

**Affiliations:** ^1^ School of Basic Medical Science, Zhejiang Chinese Medical University, Hangzhou, Zhejiang, China; ^2^ Department of Traditional Chinese Medicine, Zhejiang Cancer Hospital, Hangzhou, Zhejiang, China

**Keywords:** lung cancer, GART, cell proliferation, cell migration, PAICS

## Abstract

**Background:**

Lung adenocarcinoma (LUAD) is the primary subtype of Non-small cell lung cancer (NSCLC) and a serious threat to human health. However, the precise molecular mechanisms in lung cancer remain largely unexplored.

**Methods:**

Herein, we performed proteomic analysis in a cohort of 20 LC primary tumors and their paired normal tissues. The expression levels and prognostic value of hub proteins were also explored in LUAD using public databases. Glycinamide ribonucleotide transformylase (GART) expression was detected by qRT-PCR in LC cell lines. The roles of GART were assessed by CCK-8, colony formation, Wound healing assays, and xenograft tumor model. Expression levels of the PAICS-Akt-β-catenin pathway were estimated through qRT-PCR and western blot assays.

**Results:**

The proteomic analysis of tumor tissues of LC indicated that 263 proteins were upregulated and 194 were downregulated. Bioinformatics analysis showed that differentially expressed proteins were mainly associated with the regulation of apoptotic process and cell adhesion, PI3K-Akt signaling pathway, Purine metabolism, and Wnt signaling pathway. The expression of hub proteins EPRS, GART, HSPE1, and RPS6 was much higher in LUAD tissues than in normal tissues analyzed by the Ualcan database. Overexpression of GART represented a poor prognosis in LUAD patients. Additionally, the knockdown of GART effectively inhibited the cell proliferation and migration of LC cells both *in vitro* and *in vivo.* Mechanistically, qRT-PCR and western blot analyses suggested that GART deletion could inhibit the activation of the PAICS-Akt-β-catenin pathway *in vivo*.

**Conclusions:**

Our study indicated a tumor-promoting function of GART in LC through the regulation of the PAICS-Akt-β-catenin axis, and it may be used as a therapeutic target for NSCLC.

## 1 Introduction

Lung cancer (LC) is one of the deadliest diseases with the highest mortality rate and it is also the most common cause of tumor-related deaths (1, 2). According to the histological assessment, the main types of lung cancer are non-small cell lung cancer (NSCLC), small cell lung cancer (SCLC), mesothelioma, sarcoma, and carcinoid tumors, with SCLC and NSCLC being the most common, accounting for more than 90% of all cases (3, 4). Despite the contribution of chemotherapy and radiotherapy to the improvement of lung cancer treatment, survival rates remain low, with 5-year overall survival rates of 15% and 6% for patients with NSCLC and SCLC, respectively (5). Over-proliferation and metastatic progression play a crucial role in advanced lung cancer, leading to poor prognosis and low survival (6, 7). Therefore, understanding the molecular mechanisms of cancer metastasis remains a major clinical challenge.

Purines are an important raw material for the synthesis of DNA and RNA, and tumor cells require more purines to maintain rapid growth (8, 9). Glycinamide ribonucleotide transformylase (GART) is localized to chromosome 21 and catalyzes the 1^st^ folate-dependent transcarboxylation reaction for the *de novo* synthesis of purines from glutamine (10). Therefore, GART has received much attention as a research hotspot in cancer chemotherapy. Recently, increasing evidence suggests that the GART functions as a novel biomarker in various malignancies. For example, Cong and Liu et al. showed that higher expression levels of GART were closely connected with poor prognosis in hepatocellular carcinoma and glioma, and the GART knockdown inhibited cell proliferation of HepG2 and BEL-7404 cells, as well as the glioma cells, respectively (11, 12). Moreover, Tang et al. reported that the increased GART levels were also related to poor outcomes in colorectal cancer patients and contributed to tumor stemness by activating the Wnt/β-catenin pathway (13). More importantly, Kawamura et al. found that GART deletion inhibited the *de novo* nucleotide-dependent cell proliferation and survival of A549 cells (14). Hence, there is an urgent need to understand the molecular mechanisms of GART-promoted proliferation and metastasis in lung cancer and further identify promising therapeutic targets.

Human phosphoribosylaminoimidazole carboxylase phosphoribosylaminoimdiazole succinocarboxamide synthetase (PAICS) is a bifunctional enzyme that also participates in *de novo* purine synthesis (15, 16). Increasing evidence has suggested that a notable increase in PAICS promotes cell proliferation and migration in oral squamous cell carcinoma (17), glioma (18), and NSCLC (19). In addition, Wnt/beta-catenin signaling contributes to purine metabolism for facilitating oxaliplatin resistance in colorectal cancer (20). As we know, AKT and Wnt/β-catenin signaling pathways are associated with the proliferation and migration of human malignancies, including lung cancer (21–23). However, the effects and potential mechanisms of action of GART/PAICS-induced activation of AKT-β-catenin axis in cancer proliferation and metastasis are unclear.

In this study, we used high-throughput sequencing technology of proteomics to analyze differentially expressed proteins of Lung cancer patients’ tissues and identified the upregulated GART. GART was significantly upregulated in lung cancer cells, repressed expression of GART markedly inhibited the proliferation and migration of lung cancer cells *in vitro* and *in vivo*. More importantly, the results of bioinformatics analysis found that GART could bind to PAICS, thereby promoting the activation of the AKT/β-catenin pathway. Further mechanistic research discovered that GART deletion profoundly decreased the expression levels of PAICS and PI3K/AKT/β-catenin pathway in lung cancer. The findings of this study may offer new insights into GART as a novel biomarker and potential therapeutic target for cancer proliferation and metastasis of lung cancer.

## 2 Materials and methods

### 2.1 Clinical specimens

In total, 20 LC primary tumors and their adjacent control tissues were collected from patients who underwent surgery between March 2019 and September 2019 at Zhejiang Cancer Hospital (Zhejiang, China). The specimens were rapidly frozen in liquid nitrogen during surgery and kept in -80 °C until subsequent use. All processes were approved by the Ethics Committee of Zhejiang Cancer Hospital (Approval No. IRB-2018-219) and written consent was obtained from all study participants.

### 2.2 High-throughput label-free proteomics of LC tissues

The label-free proteomics analysis was conducted according to liquid chromatography‐high resolution tandem mass spectrometry. The protein samples were extracted from LC tissues by homogenizing in RIPA lysis buffer (Beyotime Biotechnology, China) and quantified using the BCA kit (UUbio, Suzhou, China). After reduction and alkylation, lung proteins were digested with trypsin (Promega, Madison, Wisconsin, USA) overnight at 37°C, and next, the peptides were collected using the MnoSpin C18 column (GL Sciences, Japan). Data independent acquisition (DIA)-based proteomic analysis was carried out using LC-MS/MS on Q Exactive™ HF-X (Thermo Fisher Scientific) coupled to an EASY-nLC 1000 UPLC system (Thermo Fisher Scientific) at PANOMIX Biomedical Tech Co., LTD (Suzhou, China). Protein identifications were processed using Proteome Discoverer (v2.4) and searched using the Mascot (Matrix Science, London, UK; version 2.2). The 2 folds cut-off and *P-*value <*0.05* were selected for the identification of differentially expressed proteins.

### 2.3 Bioinformatics analysis

To further investigate the roles of differentially expressed proteins, the Gene ontology (GO) of the biological process and the Kyoto Encyclopedia of Genes and Genomes (KEGG) pathway analyses were performed using Sangerbox online tool (http://sangerbox.com/) (24). For screening the hub proteins, the construction of protein-protein interaction (PPI) network was performed using the online search tool for the retrieval of interacting genes (STRING, version 12.0) database. After visualization in Cytoscape software, the top 4 hub proteins were selected according to the degree value by using the CytoHubba plugin. Additionally, the expression levels and overall survival analyses of hub proteins were explored by the UALCAN database (25). And, the correlation between PAICS and AKT and Wnt3 was analyzed by the GEPIA database (26).

### 2.4 Cell culture and transfection

The lung cancer cell lines (H522, HCC827, H1975, A549, and H1299) and human normal lung epithelial cells (BEAS-2B) were purchased from iCell Bioscience Inc (Shanghai, China) and cultured in Dulbecco’s modified Eagle’s medium (DMEM, Gibco, Carlsbad, CA, USA) with 10% fetal bovine serum (Opcel, Nei Monggol, China) in a humidified atmosphere with 5% CO_2._ For transfection, GenePharma (Shanghai, China) was responsible for designing short hairpin RNAs specifically targeting GART (shGART#1/#2) and corresponding negative controls (shNC). Cell transfection was performed using Lipofectamine™ 3000 (Invitrogen, USA) according to the manufacturer’s protocol. Finally, transfection efficiency in the A549 and H1299 cells was confirmed by quantitative real-time PCR (qRT-PCR). The A549 and H1299 cells were transfected with corresponding plasmid and NC for 48 h before further assays.

### 2.5 Cell proliferation assays

Cell proliferation was assessed by using Cell Counting Kit-8 (CCK-8, C0039, Beyotime, China) and colony formation experiments, respectively. In brief, the transfected A549 and H1299 cells (3,000 cells/well) were seeded into 96-well plates, and supplemented with 10 μL of CCK-8 solution at 24, 48, and 72 h, respectively. Next, the optical density (OD) at 450 nm was measured using a microplate reader (CMaxPlus, Molecular Devices). For colony formation assay, the transfected A549 and H1299 cells (1,000 cells/well) were inoculated into a 6-well plate with DMEM containing 10% FBS for 2 weeks. The medium was changed every 3 days. After two weeks, the cells were fixed in 4% paraformaldehyde for 60 min and then stained with 0.1% crystal violet for 15 min at room temperature. At last, the number of cell colonies were counted using a microscope (ICX41, SOPTOP, Ning Po, China).

### 2.6 Wound healing assay

To investigate the effect of GART deletion on cell motility in LC cells, we then conducted the scratch wound assay. Briefly, the transfected A549 and H1299 cells (1×10^6^ cells//well) were cultured in 6-well plates for 24h. Then, the cells were wounded by a 200 μL pipette tip. At 0 and 24 h after injury, images were photographed with the light microscope. Finally, the percentage of wound closure was calculated by using the Image J software (version 1.8.0, NIH, Bethesda, MD, USA).

### 2.7 Xenograft experiments

Male athymic BALB/c nude mice (8-12 weeks old) were purchased from SLAC Laboratory Animal Co., Ltd (Shanghai, China, SCXK(Hu)2022-0004) and housed in the specific pathogen-free (SPF) surroundings. All mice were randomly divided into shGART and shNC groups, with 8 mice in each group. A total of 3×10^6^ A549 cells with GART knockdown (shGART) and negative control cells in 0.2 ml of PBS were injected subcutaneously into the right flanks of nude mice. Tumor volume was monitored every 7 days using a vernier calipers, and calculated as: tumor length × tumor width^2^/2. After 28 days, the mice were sacrificed, and the tumor tissues were collected, weighed and also employed for histopathological examination and Western blotting.

### 2.8 Histopathological observation of tumor tissues

The tumor tissues from nude mice were fixed in 4% paraformaldehyde for 24h. Then, the tissues were dehydrated and embedded in paraffin and also cut into 4 μM thick slices. After that, the sections were stained with hematoxylin and eosin (H&E, Solarbio). The histopathological changes of tumor tissues were observed under a light microscope (Eclipse Ci-L, Nikon, Japan).

### 2.9 QRT-PCR assay

Total RNA was extracted from the LC cells and the tumor tissues by the EZ-10 Total RNA Mini-Preps Kit (B618583-0250, Sangon Biotech, Shanghai, China) and reverse transcribed into cDNA by PC70-TRUEscript RT MasterMix (OneStep gDNA Removal) (PC7002, Aidlab, Zhejiang, China). Quantitative PCR analysis was performed using a 2 x Dual SYBR Green qPCR Mix (Universal ROX) (PC6202, Aidlab) on a LightCycler^®^ 96 real-time PCR System (Roche). The primer sequences are shown as follows: GART, Forward: 5′‐AATTGGCAGTGGAGGAAGGG-3′, Reverse: 5′‐AGTTGCGACAGTATCACCCC-3′; PAICS, Forward: 5′‐GCAGGGAATGCAGCTAGGAA-3′, Reverse: 5′‐GCAGCAAACTGAGCTGATCC-3′; AKT, Forward: 5′‐CCTCAAGAACGATGGCACCT-3′, Reverse: 5′‐TAGGAGAACTTGATCAGGCGG-3′; β-catenin, Forward: 5′‐ACTGGCAGCAGCAGTCTTAC-3′, Reverse: 5′‐GGCAGCCCATCAACTGGATA-3′; β-actin, Forward: 5′‐CATGTACGTTGCTATCCAGGC-3′, Reverse: 5′‐CTCCTTAATGTCACGCACGAT-3′. β-actin was used as an internal reference gene and the expression levels were calculated using the 2^-ΔΔCT^ method (27).

### 2.10 Western blot assay

LC cells and tumor tissues were lysed with RIPA buffer (P0013B, Beyotime) containing protein phosphatase (P1260, Solarbio, Beijing, China) and protease inhibitors (P6730, Solarbio). After centrifugation, the supernatants were collected and quantified using a BCA protein assay kit (P0012, Beyotime). An equivalent amount of protein samples were separated by SDS-PAGE and transferred onto polyvinylidene difluoride (PVDF) membranes. The membranes were blocked with 5% skimmed milk for 2h, and then incubated with the following primary antibodies: PCNA (cat: 10205-2-AP, 1:20000, Proteintech, Wuhan, China), E-cadherin (cat: 20874-1-AP, 1:20000, Proteintech), MMP9 (cat: AF5228, 1:500, Affinity Biosciences, Jiangsu, China), N-cadherin (cat: 66219-1-Ig, 1:20000, Proteintech), Vimentin (cat: R22775, 1:500, ZEN-BIOSCIENCE, Chengdu, China), PAICS (cat: 12967-1-AP, 1:1000, Proteintech), Ki67 (cat: AF0198, 1:500, Affinity), p-PI3K (cat: AF3241, 1:500, Affinity), p-AKT (cat: AF0016, 1:500, Affinity), β-catenin (cat: 8480T, 1:1000, Cell Signaling Technology), and β-actin (cat: 81115-1-RR, 1:10000, Proteintech) overnight at 4°C. Next, the membranes were washed with TBST and incubated with HRP-conjugated secondary antibodies (cat: 7074, cat: 7076, 1:6000, Cell Signaling Technology) for 1 h at room temperature. Finally, the protein bands were detected by ECL reagent and analyzed using ImageJ software.

### 2.11 Statistical analysis

All results were expressed as mean ± standard deviation (SD). All data were compared by two-tailed unpaired Student’s *t-test*. *P<0.05* was considered statistically significant.

## 3 Results

### 3.1 Identification of differentially expressed proteins in tumor tissues of LC patients

**Figure 1:**
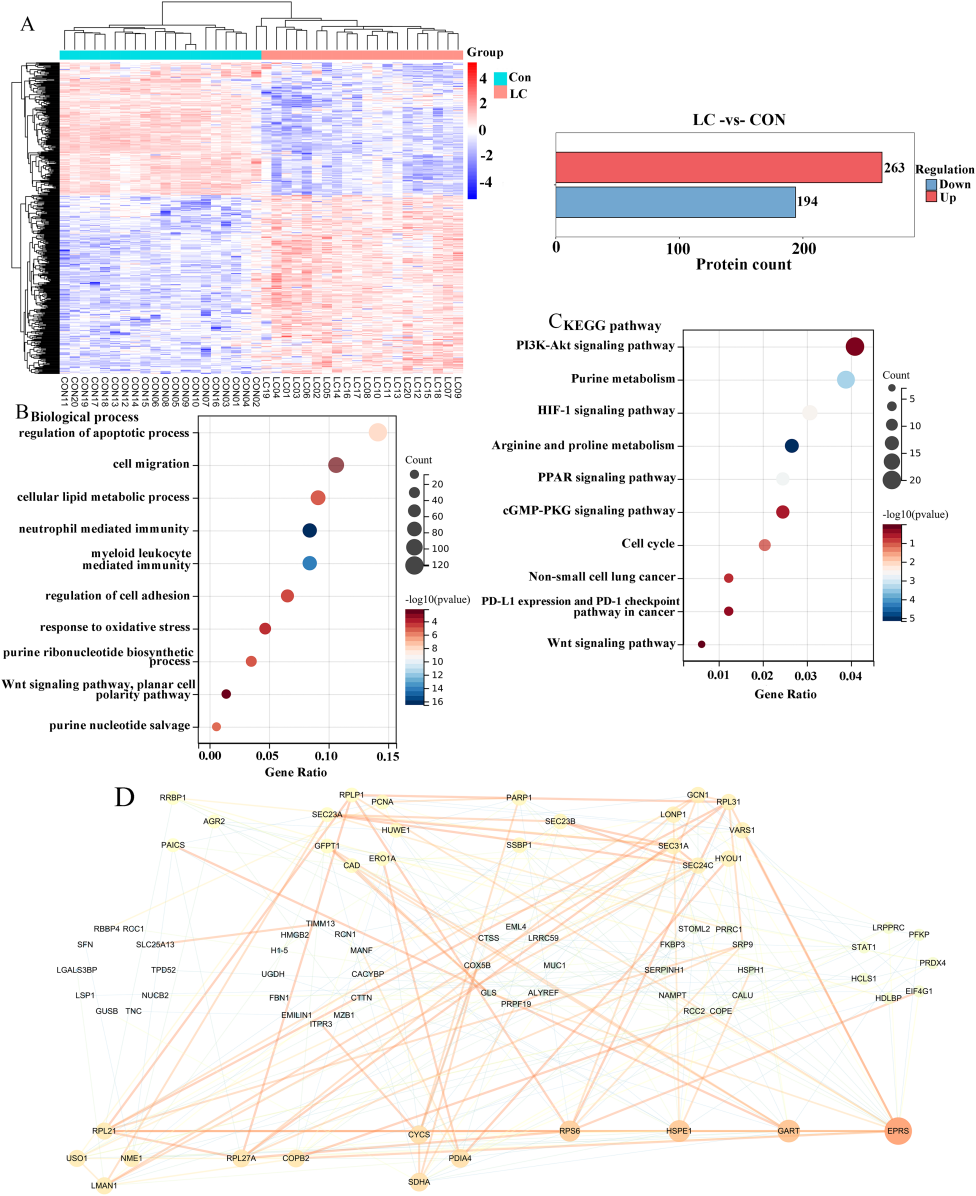
Analysis of differentially expressed proteins in LC tissues. **(A)** The heatmap plot of differentially expressed proteins between LC patients and control samples. Functional enrichment analysis of differentially expressed proteins in terms of biological process **(B, C)** Kyoto Encyclopedia of Gene and Genome (KEGG) pathways. **(D)** Construction of protein-protein interaction (PPI) network of differentially expressed proteins. Degree values were used to identify hub proteins, with the red color representing a higher degree, and the same degree of nodes formed a circle.

To further observe the changes between LC and control tissues, we analyzed all differentially expressed proteins of cancer and normal samples using proteomic analysis (28, 29). In the heat map, we obtained 457 differentially expressed proteins, of which 263 were upregulated in red, and 194 were downregulated in blue (**Figure 1A**). Next, we also conducted GO and KEGG analyses on these differentially expressed proteins. According to the GO analysis of the biological process category, differentially expressed proteins were mainly enriched in the regulation of apoptotic process, cell migration, cellular lipid metabolic process, regulation of cell adhesion, planar cell polarity pathway, and purine nucleotide salvage (**Figure 1B**). For the KEGG analysis, differentially expressed proteins were mainly enriched in the PI3K-Akt signaling pathway, Purine metabolism, HIF-1 signaling pathway, PD-L1 expression and PD-1 checkpoint pathway in cancer, and Wnt signaling pathway (**Figure 1C**). To further discover the hub proteins, all differentially expressed proteins were uploaded to the STRING database to construct the PPI network, which was analyzed using Cytoscape software. There were 80 nodes and 228 edges in the PPI network. Meanwhile, a total of 4 hub proteins, namely glutamyl-prolyl-tRNA synthetase (EPRS), GART, Heat shock protein family E member 1 (HSPE1), and ribosomal protein S6 (RPS6) were identified by degree value (**Figure 1D**), all of which were upregulated.

### 3.2 Transcription levels and prognosis of hub proteins of LUAD patients

**Figure 2:**
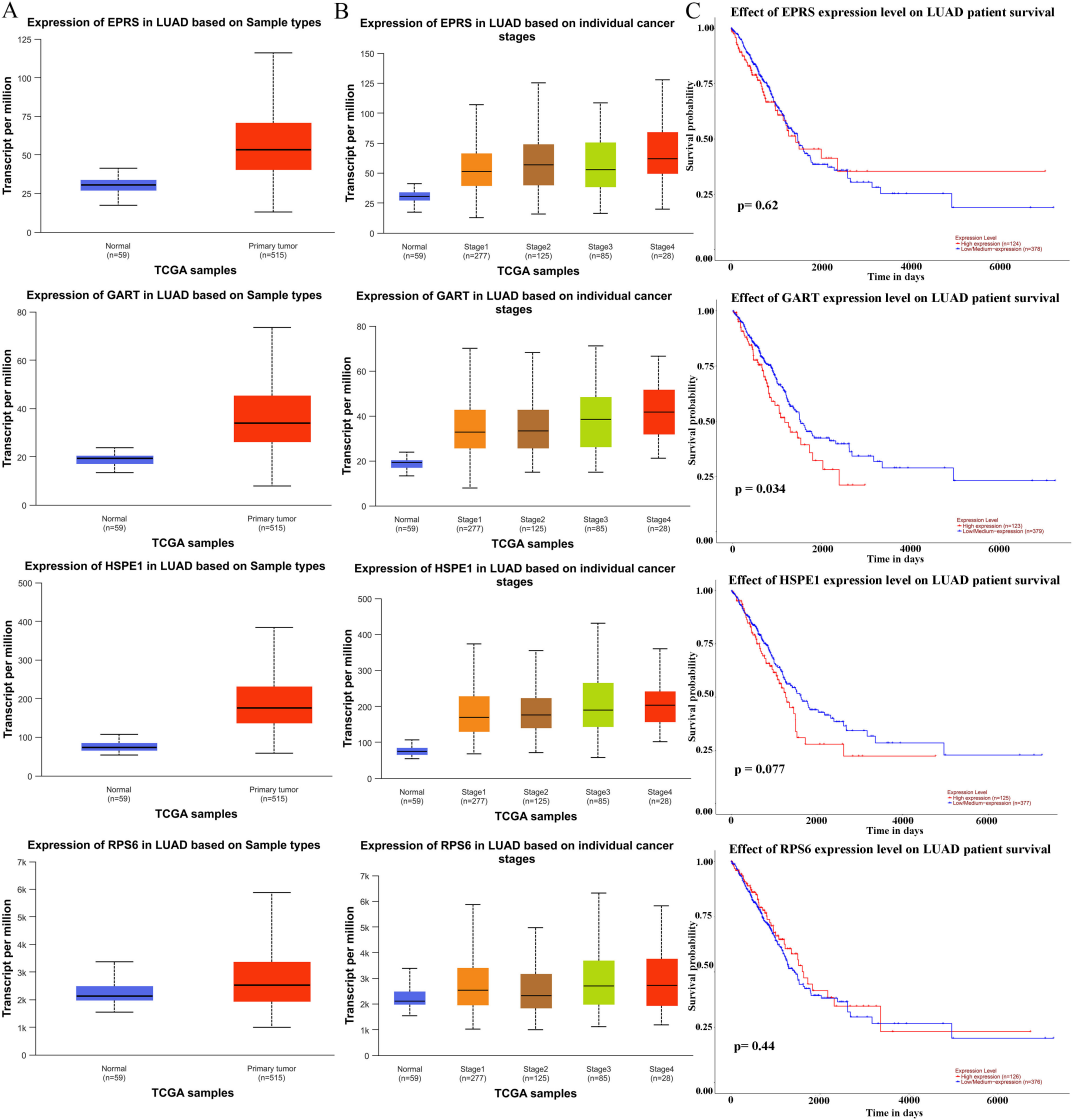
Expression levels and prognostic analysis of hub proteins in LC patients. **(A)** The expression of hub proteins (EPRS, GART, HSPE1, and RPS6) in lung adenocarcinoma (LUAD) patients in the Ualcan database. **(B)** EPRS, GART, HSPE1, and RPS6 differential expression in LUAD with individual cancer stages. **(C)** The prognostic significance of the EPRS, GART, HSPE1, and RPS6 in patients with LUAD, is based on the TCGA dataset in the Ualcan database. The red curve represented the survival probability of LUAD patients with high expression, the blue curve represented the survival probability of LUAD patients with low/medium expression.

To further determine the roles of hub proteins in LC, the UALCAN database was selected to discuss the expressions and prognostic value of the hub proteins in LUAN tissues and control lung tissues. As a result, the expression levels of EPRS, GART, HSPE1, and RPS6 were up-regulated in LUAD tissues (**Figure 2A**). As shown in **Figure 2B**, the EPRS, GART, HSPE1, and RPS6 expression levels were positively correlated with the TNM stage of LUAD. Since the expression levels of EPRS, GART, HSPE1, and RPS6 were closely associated with the progress of LUAD, we next investigated its prognosis significance. The results from the UALCAN database showed that the high expression of GART was closely correlated with poor survival in LUAD, while there were no significant differences in EPRS, HSPE1, and RPS6 in LUAD (**Figure 2C**). Hence, these results suggested that GART could be used as a prognostic marker for LC patients.

### 3.3 Downregulation of GART inhibited the cell proliferation and migration of LC cells

**Figure 3:**
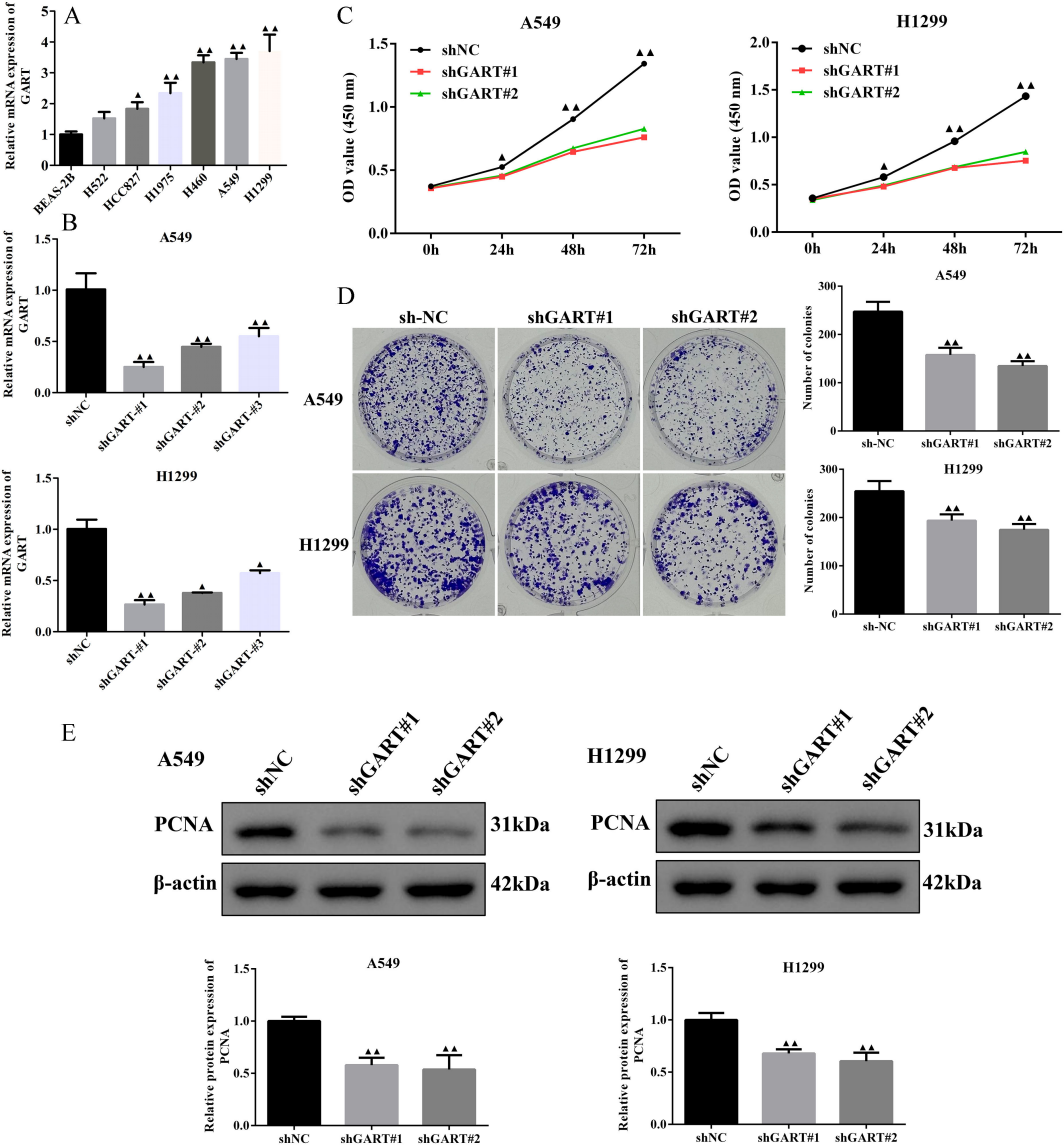
Knockdown of GART inhibited lung cancer cell proliferation *in vitro*. **(A)** Expression levels of GART in several NSCLC cell lines and BEAS-2B cells were assessed using RT-qPCR. **(B)** RT-qPCR assays were used to measure the GART levels in A549 and H1299 cells after transfection of shNC, shGART#1, or shGART#2 lentiviral vectors. **(C-D)** Cell proliferation of A549 and H1299 cells with GART knockdown was assessed using CCK-8 and colony formation assays, respectively. **(E)** The PCNA Expression levels in A549 and H1299 cells transfected with shNC, shGART#1, or shGART#2 were analyzed using Western blotting. The results represent three independent experiments. ^▲^ *P<0.05*, ^▲▲^ *P<0.01 vs.* shNC group.

**Figure 4:**
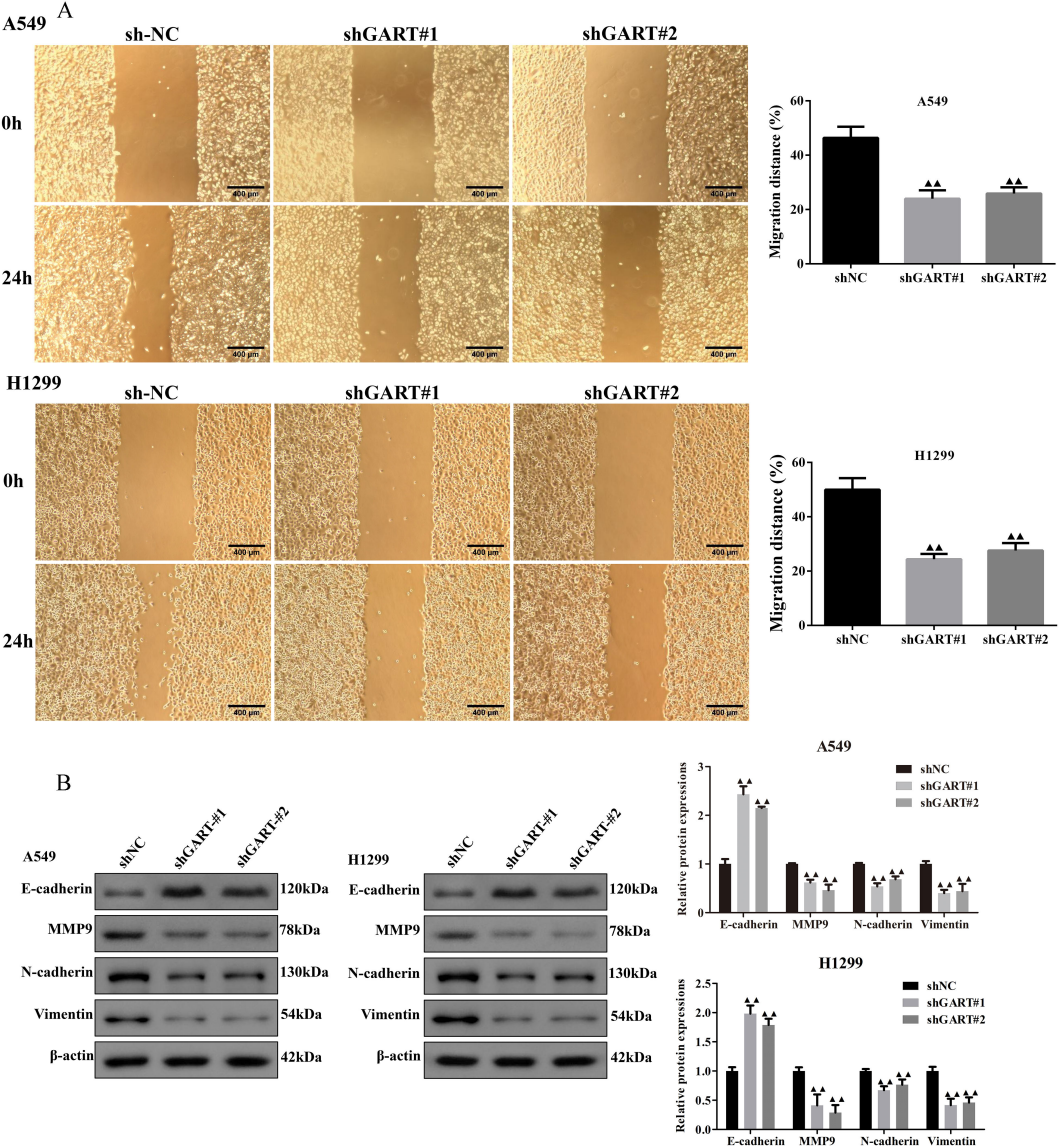
Knockdown of GART inhibited lung cancer cell migration *in vitro*. **(A)** Wound healing assay was carried out to analyze the migration ability of transfected A549 and H1299 cells. **(B)** Western blot assay was performed to measure the protein levels of epithelial-mesenchymal transition (EMT) markers (E-cadherin, MMP9, N-cadherin, and Vimentin) in transfected A549 and H1299 cells. The results represent three independent experiments. ^▲▲^ *P<0.01 vs.* shNC group.

To further unearth the biological effects of GART, qRT-PCR was used to measure the mRNA expression levels of GART in LC cell lines. The results showed that the expression of GART was significantly up-regulated in LC cells than in BEAS-2B cells, especially in A549 and H1299 cells that then were selected to conduct further experiments (**Figure 3A**). Subsequently, we first established the GART knockdown cell lines by using shRNAs in A549 and H1299 cells, respectively. As shown in **Figure 3B**, the expression levels of GART were significantly decreased in A549 and H1299 cells. Next, cell viability was assessed in each group by CCK-8. The results showed that knockdown of GART observably inhibited cell viability (**Figure 3C**). Similarly, colony formation was significantly reduced in A549 and H1299 cells after GART knockdown (**Figure 3D**). Also, we found that the protein expression levels of PCNA in A549 and H1299 cells significantly decreased after the transfection of GART shRNAs (**Figure 3E**). Furthermore, GART on the migration of LC cells was measured by wound healing assay. As shown in **Figure 4A**, the knockdown of GART could significantly inhibit the migration rate of A549 and H1299 cells. Besides, the biomarkers of EMT were detected in GART knockdown cells by using Western blot. Results showed that knockdown of GART significantly increased the expression of E-cadherin, and suppressed the expression of MMP9, N-cadherin, and Vimentin (**Figure 4B**). Taken together, these data indicate that GART could promote cell proliferation and migration of LC cells.

### 3.4 Downregulation of GART restrained tumor growth *in vivo* by regulating PAICS-Akt-β-catenin pathway

**Figure 5:**
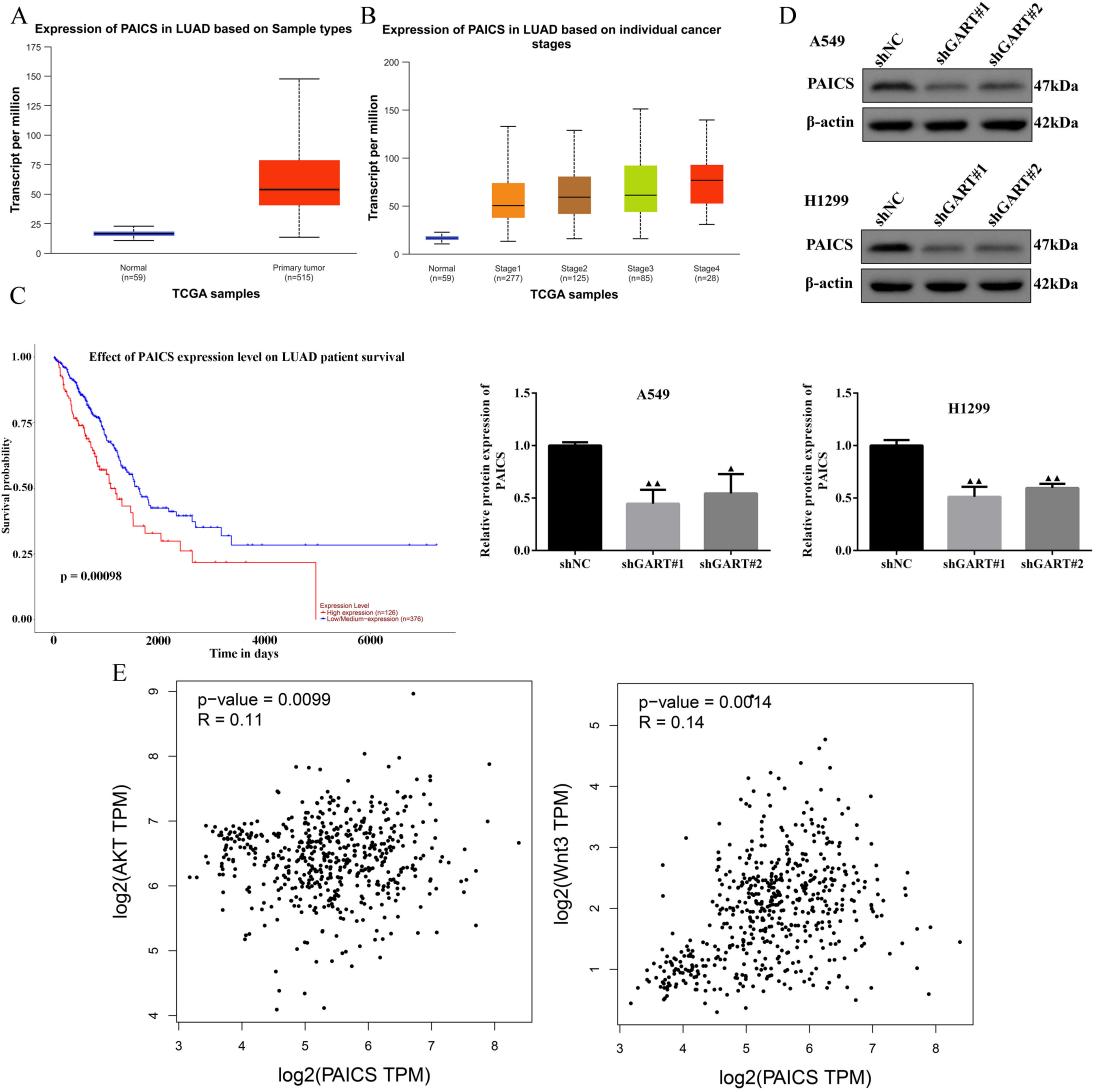
Expression levels of PAICS and the relationship among GART, PAICS, AKT, and Wnt. **(A-B)** PAICS expression was evaluated in LUAD patients based on sample types and individual cancer stages using the UALCAN database. **(C)** The UALCAN database was used to illustrate the influence of high PAICS expression on the prognosis of LC patients. **(D)** The expression of PAICS was measured by western blot. **(E)** Scatterplot of the correlation between PAICS and AKT and Wnt3 acquired from the GEPIA database.

**Figure 6:**
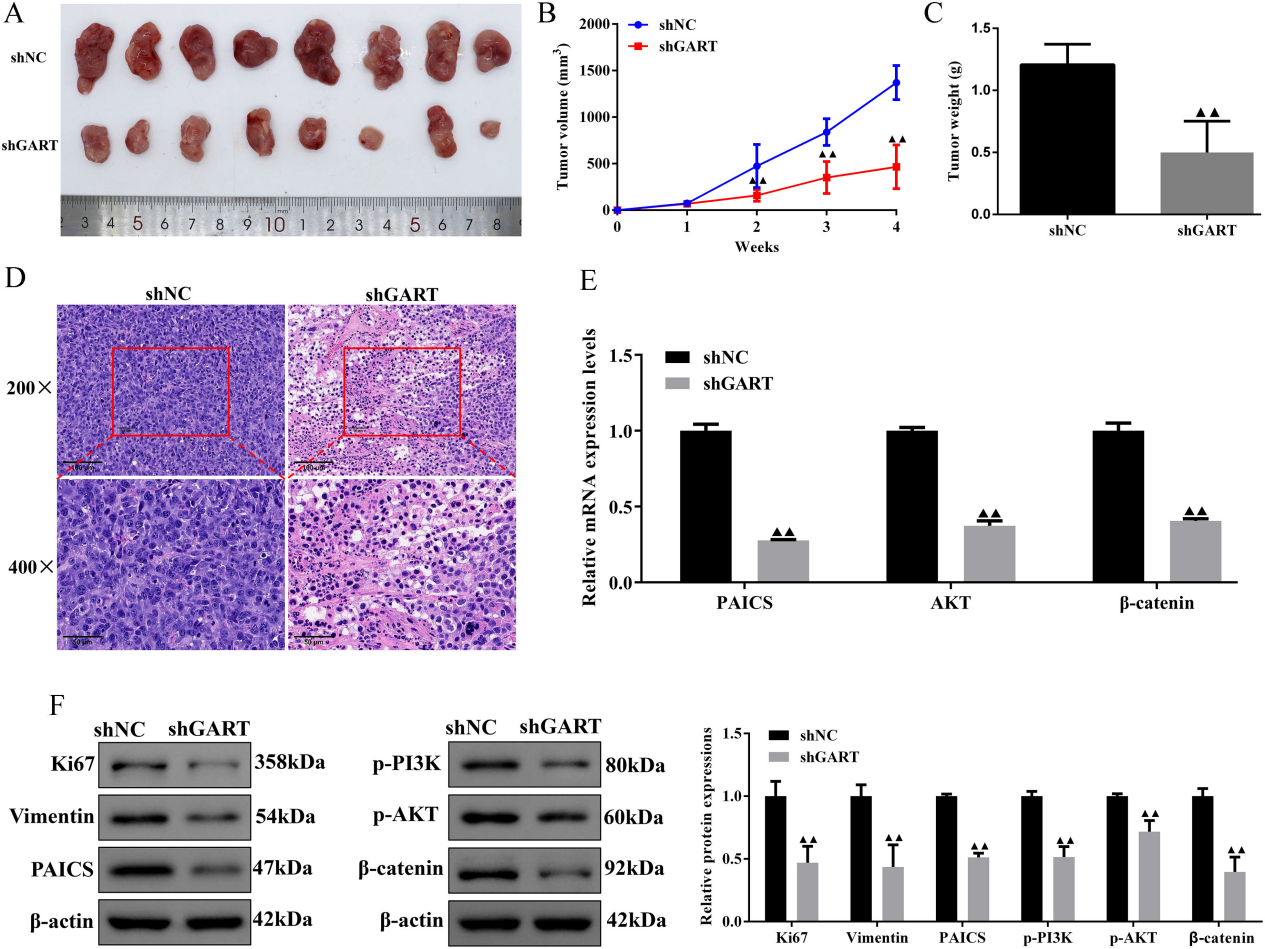
Knockdown of GART inhibited tumor growth in mice treated with A549 cells transfected with shNC or shGART plasmid. **(A)** Representative tumor images of shNC and shGART subcutaneous xenograft models. **(B-C)** The effect of GART knockdown on tumor volume curve and tumor weight were analyzed. **(D)** Histopathological changes of tumor tissues were observed using HE staining. Scale bar, 50 μm, 100 μm. **(E)** Expression levels of PAICS, AKT, and β-catenin in tumor tissues were assessed by RT-qPCR assays. **(F)** Expression levels of Ki67, vimentin, PAICS, p-PI3K, p-AKT, and β-catenin in tumor tissues were assessed by Western blot assays. Data were expressed as mean ± SD (n=8). ^▲▲^ *P<0.01 vs.* shNC group.

In the PPI network, we found that GART was connected to PAICS with a high degree value. For this point of view, we firstly investigated the expression levels and prognosis of PAICS. The UALCAN database showed that PAICS transcription levels were significantly higher in tumor tissues of LUAD patients than in adjacent normal tissue, as accompanied by an elevation in TNM stage (**Figures 5A, B**). Also, higher PAICS expression was related to poor overall survival in LUAD (**Figure 5C**). These results illustrated that PAICS expression closely associated with the prognosis of LUAD. Furthermore, the results of Western blotting showed that GART knockdown inhibited the protein expression levels of PAICS in A549 and H1299 cells (**Figure 5D**). Notably, we further found that there was significant correlation between PAICS expression and AKT and Wnt3 expressions in LUAD in the GEPIA database (**Figure 5E**). Subsequently, we assessed the function of GART knockdown on the tumor growth *in vivo* by using A549 tumor-bearing mouse model. As expected, the results showed that mice in the GART knockdown group had remarkably smaller tumor volumes and weight compared to those in the shNC group (**Figures 6A–C**). H&E staining revealed a significant presence of tumor cells in the shNC group. In contrast, the number of tumor cells was reduced in the shGART group accompanied by a small amount of necrosis (**Figure 6D**). The results of qPCR assays indicated that the mRNA levels of PAICS, AKT, and β-catenin were significantly reduced in the shGART group (**Figure 6E**). Besides, Western blot assays showed that the protein expression levels of Ki67, Vimentin, PAICS, p-PI3K, p-AKT, and β-catenin were significantly decreased in tumor tissues of the shGART group compared with the shNC group (**Figure 6F**). Taken together, these findings demonstrated that GART promoted the proliferation and migration of LC *in vitro* and *in vivo*, at least partly through the activation of PAICS-Akt-β-catenin pathway.

## 4 Discussion

With the advances in the field of high-throughput sequencing, it enhance our understanding of the molecular mechanisms of lung cancer, thus also providing new ideas for the diagnosis and treatment of lung cancer (30). In the current study, we identified 263 differentially upregulated proteins and 194 differentially downregulated proteins between LC samples and corresponding non-tumor normal tissues by analyzing the proteomics. The functional enrichment analysis of these differentially expressed proteins suggested that central pathways and hub proteins might give rise to novel insights into the molecular mechanisms managing LC progression.

In this study, GO enrichment analyses showed that differentially expressed proteins were mainly enriched in the regulation of apoptotic process, cell migration, cellular lipid metabolic process, regulation of cell adhesion, planar cell polarity pathway, and purine nucleotide salvage, as well as those biological events in the tumor tissues were related to cell proliferation. More importantly, purines and pyrimidines are cornerstones of DNA synthesis, and due to the rapid proliferation of tumor cells, they have an enhanced need for nucleotide metabolism, whereas extensive activation and exploitation of the nucleotide *de novo* synthesis pathway (31). Thus, the role of activation of purine metabolic pathways in tumor progression has attracted attention. Moreover, the differentially expressed proteins were also found to be enriched in the PI3K-Akt signaling pathway, Purine metabolism, and Wnt signaling pathway. Additionally, it has been verified that the unbalance of PI3K-Akt and Wnt pathways is a critical stimulus for the advancement of most malignancies (32–34). This implies that the differentially expressed proteins associated with purine metabolism might participate in cell proliferation and migration of LC by regulating PI3K-Akt and Wnt signaling pathways.

Of significance, the top 4 hub proteins were selected in the PPI network. Next, we investigated the transcriptional expression and prognostic values of these hub proteins in the UALCAN database. The results showed that EPRS, GART, HSPE1, and RPS6 expressions were significantly increased in LC with the stage of malignant progression. EPRS is a member of the aminoacyl-tRNA synthetases (AARSs) superfamily that catalyzes the aminoacylation of glutamate and proline tRNAs, is responsible for the translation of the genetic code, and is involved in protein synthesis (35). At present, the function of EPRS in lung carcinogenesis is largely unknown. However, Liu et al. reported that EPRS helps in the development of gastric cancer by enhancing the WNT/GSK-3β/β-catenin pathway (36). Also, the EPRS-ATF4-COLI axis could be used as a promising biomarker and therapeutic target for anaplastic thyroid carcinoma (37). HSPE1 is a chaperone protein that plays an important role by preserving normal mitochondrial functions and cellular metabolism (38). In addition, Xie et al. found that HSPE1 contributed to cell proliferation, migration, and invasion in LUAD through the increasing aerobic glycolysis pathway (38). Phosphorylation and/or overexpression of RPS6, a component of the small 40S ribosomal subunit, were noticed to participate in multiform malignancies (39–41). Interestingly, Chen et al. demonstrated that RPS6 knockdown restrained tumor growth of NSCLC via mediating G0-G1 cell cycle arrest (41). The inhibitor of GART, which serves as a major enzyme in nucleotide metabolism, has been shown to have both cytotoxic and cytostatic effects on several types of cancer cells (42, 43). In further analysis of the prognosis, we found that GART had poor overall survival in LUAD patients. Therefore, we used GART as a key target in the diagnostic and therapeutic process of LC to provide new ideas for LC treatment. In this study, we demonstrated that knockdown of GART could inhibit the proliferation of A549 and H1299 cells by CCK-8 and cell clone formation assays. In addition, metastasis is another hallmark malignant phenotype of cancer cells. The wound healing assay demonstrated that knockdown of GART inhibited the migration of A549 and H1299 cells. Furthermore, we found that GART knockdown suppressed xenograft growth in mice. These findings suggested the potential clinical application of molecular treatment targeting GART in LC.

As for PAICS, a purine biosynthetic enzyme that catalyzes the *de novo* synthesis of DNA and is involved in the proliferation, migration, growth, and invasion of tumor cells (16, 44). Moreover, PI3K-AKT and Wnt/β-catenin axes are key signaling pathways in cancer development, and inhibition of these pathways is one of the main strategies for targeted cancer therapy (45, 46). It can be inferred that GART is positively associated with PAICS in the PPI network. In accordance with these studies, PAICS was found to be positively associated with AKT and Wnt3 expressions in patients with LUAD. Of note, we also found that the knockdown of GART significantly inhibited the PAICS expressions and the PI3K/AKT/β-catenin pathway *in vivo*. These may clarify the importance of GART in promoting cancer proliferation and migration by targeting the PAICS-Akt-β-catenin pathway in LC.

In summary, the current study showed the oncogenic effect of GART in lung cancer by promoting cell proliferation and migration *in vivo* and *in vitro* by targeting the PAICS-Akt-β-catenin pathway. Nonetheless, additional studies in both fundamental and clinical research are required to verify the molecular mechanisms.

## Data Availability

The original contributions presented in the study are included in the article/supplementary material. Further inquiries can be directed to the corresponding authors.
